# Simple and effective deposition method for solar cell perovskite films using a sheet of paper

**DOI:** 10.1016/j.isci.2021.103712

**Published:** 2021-12-31

**Authors:** Nazila Zarabinia, Giulia Lucarelli, Reza Rasuli, Francesca De Rossi, Babak Taheri, Hamed Javanbakht, Francesca Brunetti, Thomas M. Brown

**Affiliations:** 1 Department of Physics, Faculty of Science, University of Zanjan, Zanjan, Iran; 2 CHOSE (Centre for Hybrid and Organic Solar Energy), Department of Electronic Engineering, University of Rome Tor Vergata, Rome, Italy

**Keywords:** Electronic materials, Energy materials, Surface science

## Abstract

Most laboratories employ spin coating with application of antisolvent to achieve high efficiency in perovskite solar cells. However, this method wastes a lot of material and is not industrially usable. Conversely, large area coating techniques such as blade and slot-die require high precision engineering both for deposition of ink and for gas or for electromagnetic drying procedures that replace, out of necessity, anti-solvent engineering. Here we present a simple and effective method to deposit uniform high-quality perovskite films with a piece of paper as an applicator at low temperatures. We fabricated solar cells on flexible PET substrates manually with 11% power conversion efficiency. Deposition after soaking the sheet of paper in a green antisolvent improved the efficiency by 82% compared to when using dry paper as applicator. This new technique enables manual film deposition without any expensive equipment and has the potential to be fully automated for future optimization and exploitation.

## Highlights

• New method for depositing perovskite films with a piece of paper is demonstrated
• Soaking paper applicator in antisolvent boosts efficiency of solar cells by 82%
• Paper possesses right porosity and smoothness for deposition of high quality films
• Flexible perovskite solar cell efficiency made manually via paper applicator is 11%

## Graphical abstract

**Figure:**
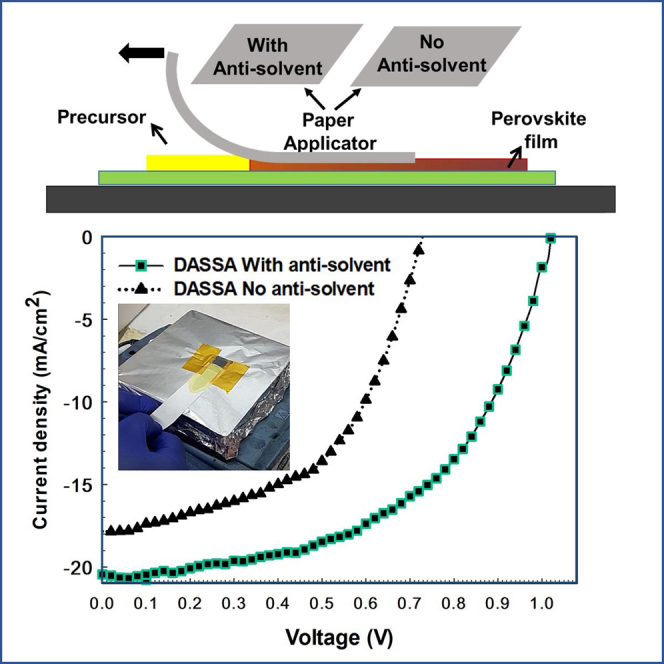

## Introduction

The deposition process of perovskite films has great influence on device performance as well as on meeting industrial goals such as scalability (Ling et al., 2021). In solution processing, crystallization starts during solvent evaporation, which is strongly dependent on the deposition technique used (Qiu et al., 2018). Spin coating is the main method to fabricate small laboratory cells and relies on fast solvent evaporation of the precursor formulation through centrifugal forces that cause solvent removal and thinning of the film. Addition of drops of antisolvent during spinning influences the morphology of the perovskite material very positively leading to high quality films and significantly enhanced efficiency in solar cells (Nie et al., 2015; Arain et al., 2019). When using a dimethyl sulfoxide (DMSO)/dimethyl formamide (DMF) solvent mixture, the removal of the lower boiling point carrier solvent (i.e., DMF) by the antisolvent determines the formation of an intermediate state (Liu et al., 2019; Yang et al., 2018). Subsequently, a complete perovskite phase layer is formed during thermal annealing (Kim et al., 2019). The uncontrolled synthesis of perovskite without antisolvent typically produces a wide variation in film morphology and grain dimension, yielding non-homogeneous films or even pinholes, which negatively affect the photovoltaic performance of solar cells (Konstantakou et al., 2017). Thus the antisolvent strategy has become a mainstay for boosting efficiency in the fabrication of solar cells via the spin coating technique.

Appropriate crystallization has also been achieved with other new methods, including soft-cover deposition in which, by removing a cover over the wet film, evaporation is accelerated (Zhong et al., 2018; Ye et al., 2016, 2017a). Nevertheless, it is slot-die coating, blade coating, inkjet, and spray coating which are considered the prime candidates to replace spin coating to move from laboratory fabrication toward industrial large-scale production (Howard et al., 2019; Li et al., 2019a, 2021a; Razza et al., 2016; Babayigit et al., 2018; Angmo et al., 2021; Cotella et al., 2017; Castro-Hermosa et al., 2021; Parvazian et al., 2019; Calabrò et al., 2018). With these linear deposition techniques, the application and removal of antisolvent become problematic (Galagan et al., 2018; Zuo et al., 2018). Apart from Kim et al. (Kim et al., 2020) who immersed the perovskite film into a container filled with antisolvent after being gravure printed (with question marks on the large amount of antisolvent required as well as it industrial practical applicability and takt times), historically the antisolvent method has been applied successfully to the spin coating process only. Heating the substrate (150-210°C) or forcing a flow of gas on the wet perovskite layer for drying are, by far, the most utilized strategies that have been implemented to control solvent evaporation and layer morphology in blade and slot-die coating (Kim et al., 2018; Brinkmann et al., 2019; Castro-Hermosa et al., 2021). With drying procedures, control over evaporation and over film morphology is difficult to achieve especially on large areas because of thermal convection effects and high precision engineering is required (Ye et al., 2017b; Zhong et al., 2018). It is apparent that the application of antisolvent procedures integrated with the deposition process and compatible with large areas has yet to be thoroughly considered and implemented.

Here, we developed a new technique we term Deposition via an Antisolvent Soaked Applicator (DASSA) for the perovskite precursor solution on a heated substrate, in which both the use of a sheet of paper for coating the perovskite layer as well as soaking the applicator in an antisolvent are novel. Furthermore, in this method, where the applicator is soaked in antisolvent, rather than the perovskite film bathed in it, the amount of solvent is reduced, and no gas flow is required. DASSA is a potentially scalable new method that combines a soft applicator that can absorb antisolvents to produce uniform pinhole-free perovskite films. The antisolvent used was ethyl acetate which is considered a green solvent (Zhang et al., 2019, 2020a), acts as a moisture absorber, protecting sensitive perovskite from airborne water during film formation and annealing. Processing was carried out at temperatures below 150° compatible with the manufacturing of flexible cells on plastic substrate (Di Giacomo et al., 2016a). Flexible perovskite solar cells (FPSCs) are particularly interesting for large area techniques because they can enable industrial roll-to-roll manufacturing as well as enabling the application to curved surfaces for both outdoor and indoor utilization (Lucarelli et al., 2017; Castro-Hermosa et al., 2020).

## Results and discussion

### Deposition and film formation

#### Deposition process and effect of different soft applicator materials

**Figure 1:**
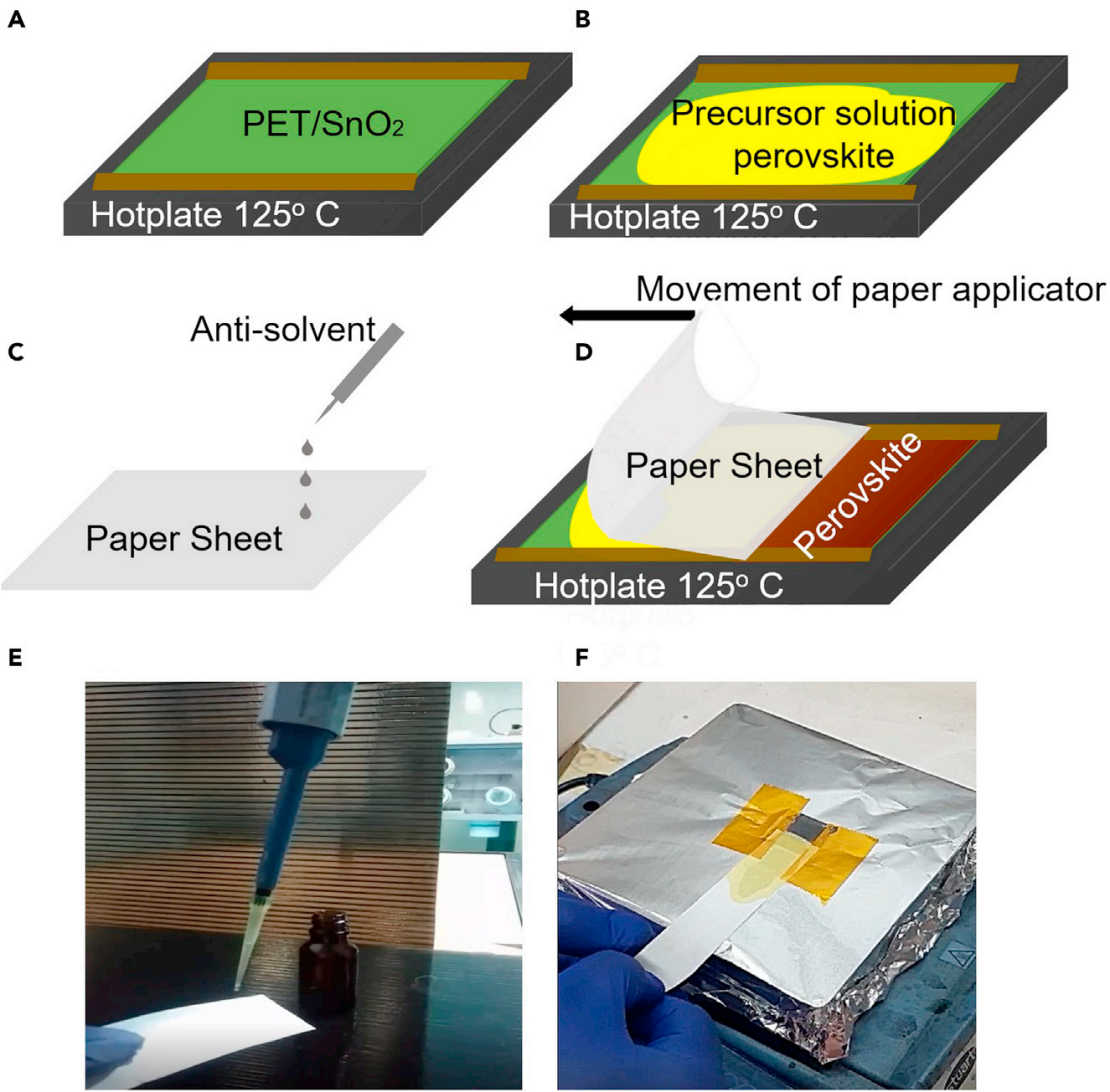
Schematic illustration of the deposition of perovskite films via an antisolvent soaked applicator (DASSA) method (A) The PET/ITO/SnO_2_ substrate with two parallel segments made of tape at the edges (the substrate is placed on a hot plate); (B) spreading of the perovskite precursor on PET/ITO/SnO_2_ substrate; (C) soaking of the piece of paper applicator in antisolvent; (D) drawing of the paper applicator over the perovskite precursor solution and tape separators from one side of the substrate to the other; (E) photograph of application of antisolvent on the piece of paper; (F) photograph of soaking the piece of paper applicator in Antisolvent and the DASSA method in which the piece of paper previously soaked in antisolvent is drawn over the substrate, with the brown-coloured perovskite film being formed at the trailing edge of the applicator. See Video S1 in supporting information for the full procedure.

**Figure 2:**
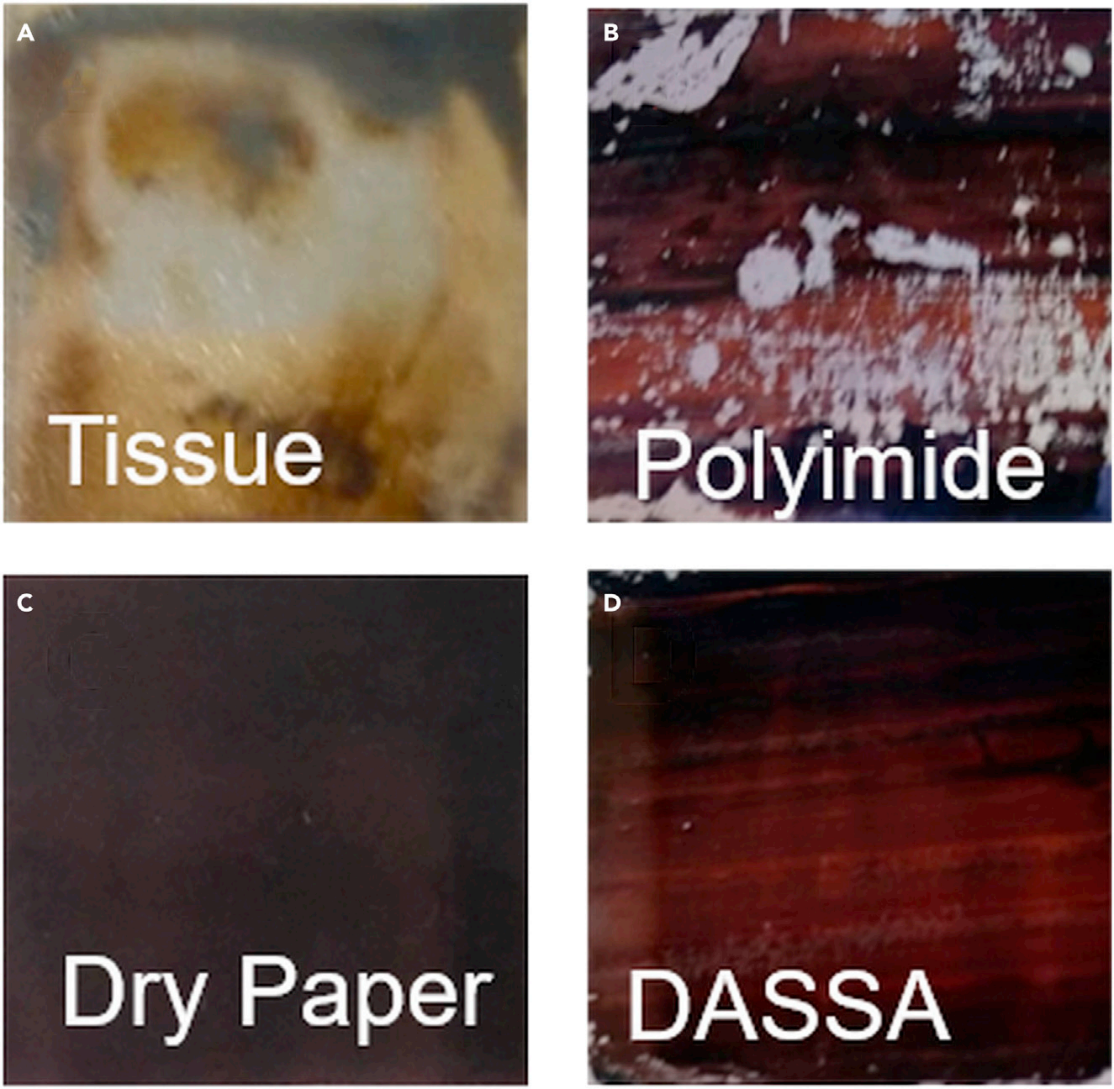
Images of perovskite films Photographs of perovskite films deposited via (A) a tissue, (B) a polyimide film, (C) a dry piece of paper, and (D) antisolvent soaked paper applicator (DASSA) on PET/ITO/SnO_2_ substrates.

We developed a deposition technique based on an applicator consisting of a simple sheet of paper (a standard laboratory paper with an 80 μm thickness, area 6 × 4 cm^2^, and weight of 0.177 g) as illustrated in Figure 1. We attached the transparent conducting substrate, on the edges of which two stripes of 50μm thick separator tape were applied, on a heated hotplate set at a fixed temperature (see Figure 1A). When the substrate was still on the hotplate, we dropped and spread double cation perovskite precursor solution via the pipette to cover the whole substrate (Figure 1B). Then the end of a piece of paper, which was soaked in ethyl acetate antisolvent, was let to rest from a height of 2-3 cm on the edge tape separators and drawn over the liquid perovskite precursor. The paper applicator was pulled manually from one side to the other of the substrate at a speed of ∼0.1 cm/s with no additional pressure apart from that exerted by the weight of the paper applicator. The color of the paper applicator during the procedure turned from its initial white to the yellowish color of double cation perovskite precursor solution, evidence of the latter being partly absorbed by the paper applicator. The drawing of the piece of paper from one end of the substrate to the other produced a thin wet film at the trailing end of the applicator. The remaining solvent evaporated after seeing air and a thin solid film was formed that turned a uniform dark brown (we advise readers to watch the Video S1 in supporting info for visual aid to this method). Figures 2C and 2D illustrate typical images of perovskite thin films characterized by high uniformity and no macroscopic pinholes and defects, which were fabricated via the DASSA methods with antisolvent and without antisolvent (i.e., only dry paper). We tried different applicators such as polyimide film, tissue, and normal paper. Very absorbing tissue paper absorbed almost all of the perovskite solutions during application leaving little film remaining over the substrate (see Figure 2A). Polyimide, on the other hand, is not able to absorb solvents, so all evaporation occurs after its removal and its movement caused the precursor solution to move away from the substrate creating inhomogeneity such as those shown in Figure 2B. The sheet of paper instead possessed the right solvent-absorbing capacity for obtaining uniform films of perovskite semiconductor. Perovskite film formation is impacted by the environment so that in air crystal growth is accelerated and introduces anisotropies in crystal morphology (Angmo et al., 2019). If the process of deposition were carried out in an inert environment, as we did for the spin-coated samples, it may be possible to obtain higher quality perovskite crystals. Nevertheless, as it is, the DASSA fabrication performed in air using a green antisolvent is more impactful for processing in normal and easier environmental conditions (Zhang et al., 2020a).

#### Effect of substrate temperature during application

**Figure 3:**
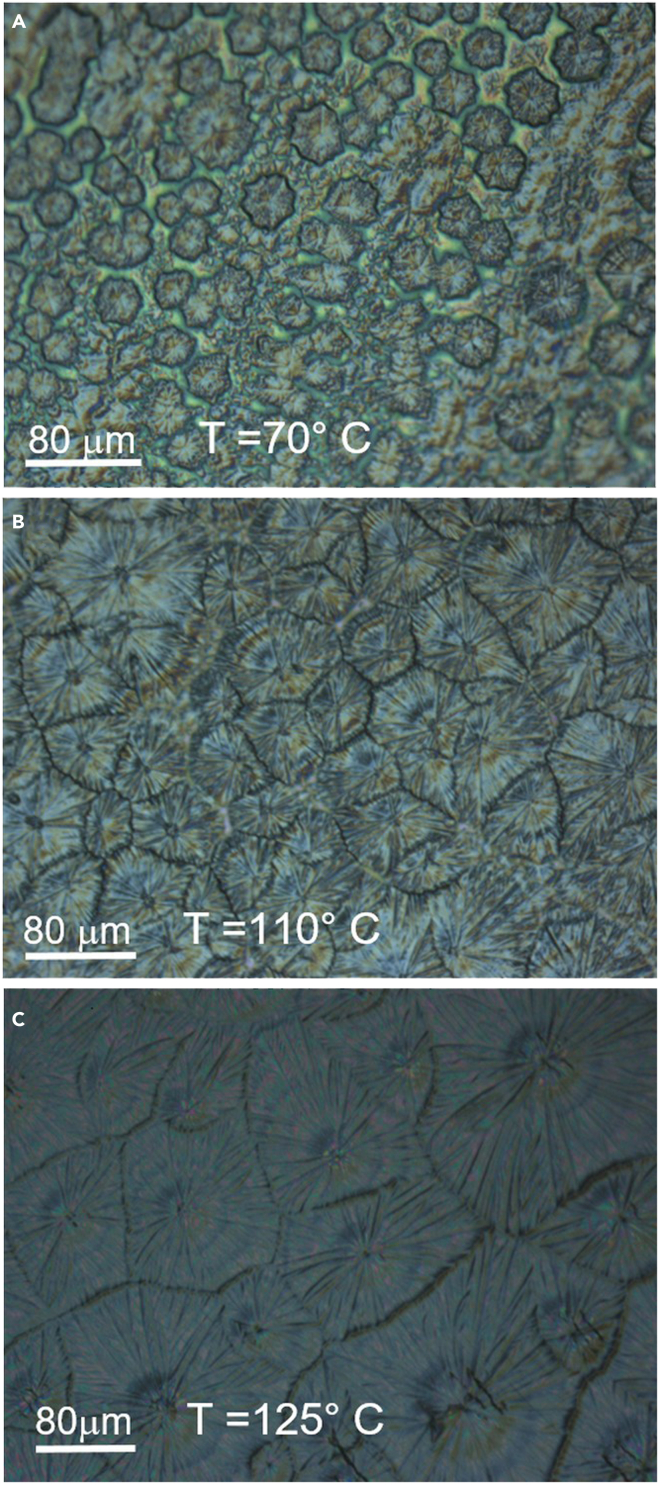
Optical micrographs of PET/ITO/SnO_2_/perovskite films (A) the perovskite precursor was deposited via the antisolvent soaked applicator (DASSA) technique at temperatures of 70°, (B) 110°, and (C) 125°(C)

The morphology of the DASSA film was significantly affected by the temperature of the substrate upon application of the film. As visible from Figure 3, at 70°C the slow drying leads to non-uniform films. Drying is faster, as well as films more uniform, at an intermediate temperature of 110°C. At 125°C, which is still a suitable temperature that does not deform flexible PET substrates (Zardetto et al., 2011), uniformity appears to be very good. From absorption measurements (Figure S1) it is estimated that the thicknesses at 70°C, 110°C, and 125°C were about 300 nm, 540 nm, and 580 nm, respectively. For the fabrication of the remainder of perovskite films in this work we opted for a temperature of 125°C.

#### Effect of soaking the paper applicator in antisolvent

**Figure 4:**
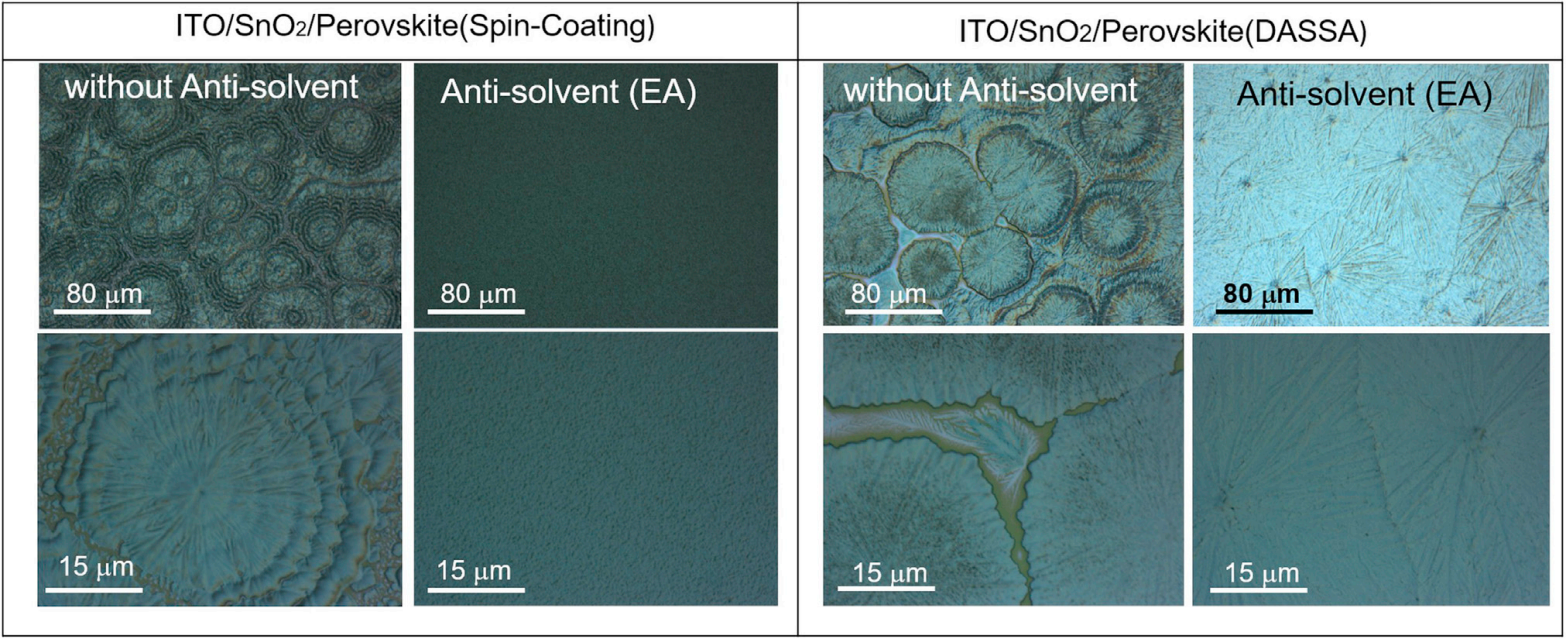
Optical micrographs of perovskite films on PET/ITO/SnO_2_ in 125°C by spin coating and deposition via an antisolvent soaked applicator (DASSA) without and with antisolvent (ethyl acetate, EA)

Figure 4 shows the optical micrographs of DASSA perovskite films with and without soaking the paper applicator in antisolvent at 125°C, together with films deposited by spin coating, again with and without the antisolvent procedure. The spin coated perovskite film without antisolvent shows non-uniform morphology with ring-like structures (Li et al., 2019b; Chen et al., 2018; Deng et al., 2015). Adding antisolvent at the end of the spin coating procedure improved the morphology and homogeneity of the spin coated perovskite film significantly. Antisolvent addition decreases the solubility of the precursor in its carrier solvent accelerating the removal of residual lower boiling point solvent (DMF), which improves nucleation and leads to a homogeneous film (Pascoe et al., 2017; Hu et al., 2017, 2020; Ye et al., 2017a).

The perovskite film deposited with just a dry piece of paper shows low uniformity and concentric rings. As with the case of the spin coating procedure, it is clear from the optical micrographs of Figure 4 that the antisolvent addition to the applicator leads to a remarkable improvement in uniformity and high-quality films are obtained.

Figure S2 shows the absorbance spectra of perovskite films deposited by spin coating and DASSA coating with and without antisolvent for both deposition methods. The absorbance indicates that adding antisolvent yields thicker layers on top of yielding better quality (Figure 4). The ring-like structures are visible in both the spin coating (no antisolvent) technique and DASSA without antisolvent, whereas upon addition of antisolvent the films become much more uniform in both the spin coating technique and the DASSA technique. The antisolvent procedure modifies the drying behavior, accelerating solvent evaporation, and the crystallization process (Kim et al., 2020).

**Figure 5:**
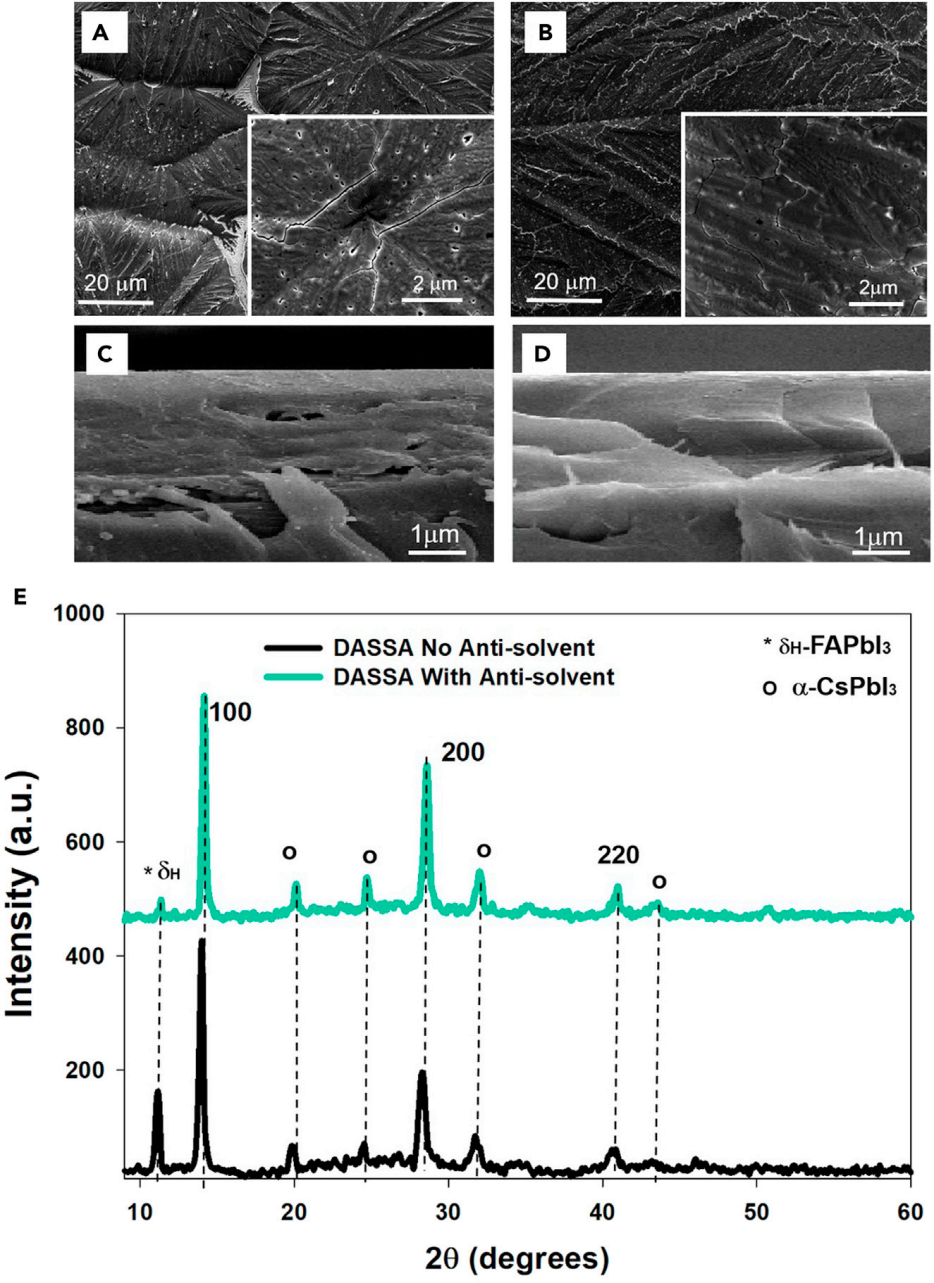
SEM images and X-ray diffraction (XRD) pattern of DASSA films with and without antisolvent (A) SEM images of perovskite films deposited with paper applicator without and (B) with antisolvent soaking of the applicator. (C) Cross section of perovskite films deposited with the DASSA method without and (D) with antisolvent. The sample structure is PET/ITO/SnO_2_/perovskite. (E) X-ray diffraction (XRD) pattern of DASSA films with and without antisolvent

SEM images in Figure 5 A, B demonstrate that soaking of the paper applicator in antisolvent gives rise to much more uniform perovskite films, even microscopically, compared to the case without antisolvent. The film created by DASSA method without antisolvent at the 20μm scale shows large structures separated by gaps which are not well interconnected. With antisolvent, instead, the structures become well-connected: considerably higher overall film uniformity is achieved (Li et al., 2021b). Wide gaps or cracks are also visible in the former case at the higher 2μm resolutions and not when soaking the applicator in antisolvent. Cross-sectional SEM images show high uniformity of the vertical phase for the DASSA case (Figure 5D) whereas for the films deposited without antisolvent a slightly more layered structure with the presence of some gaps in the film is noticed. The more uniform and better-interconnected films throughout the film's thickness for the DASSA films allow for the photo-generated carriers to propagate through the films with less defects which will lead to better performance in solar cells.

To obtain a better understanding of the influence of the DASSA method on the perovskite film and on the performance of solar cells, XRD measurements were performed (Figure 5E). The (100) and (200) reflections of the main peaks of double cation perovskite films were near 13.5°–15° and 28°–30° (Rehman et al., 2017) We report the relative height, full width at half maximum FWHM, and d-spacing for three peaks in Table S1. The full width at half maximum (FWHM) of the (100) diffraction peak is reduced from 0.36 to 0.28 when using the antisolvent soaking. An even more significant narrowing also occurs for the 200 and 220 crystal peaks, a sign that CsPbI_3_ crystals form in black phase, which are both also more intense (Ono et al., 2017). The narrowed XRD signal can be related to enhanced crystallinity in the direction perpendicular to the substrate with fewer crystal defects (Lohmann et al., 2020; Tung et al., 2009; Muthukumaran and Gopalakrishnan, 2012; Tsai et al., 2018). XRD can also tell us interesting information on composition. The intensity of the non-perovskite hexagonal δH-phase of FAPbI_3_ for the film deposited without antisolvent is more intense. This particular phase is non-photoactive (Li et al., 2016). The shift of the (100) reflection from 13.99° for DASSA without antisolvent to 14.14° for DASSA with antisolvent identifies a more effective substitution of Cs in Cs-FA perovskite in creating the desired phase of the double cation perovskite structure (Li et al., 2016). All the evidence points to films with better crystallinity, uniformity, and photo-active structural/chemical composition for the DASSA films deposited with the applicator soaked in antisolvent.

### Solar cells fabricated with perovskite films deposited via antisolvent soaked paper applicator

**Figure 6:**
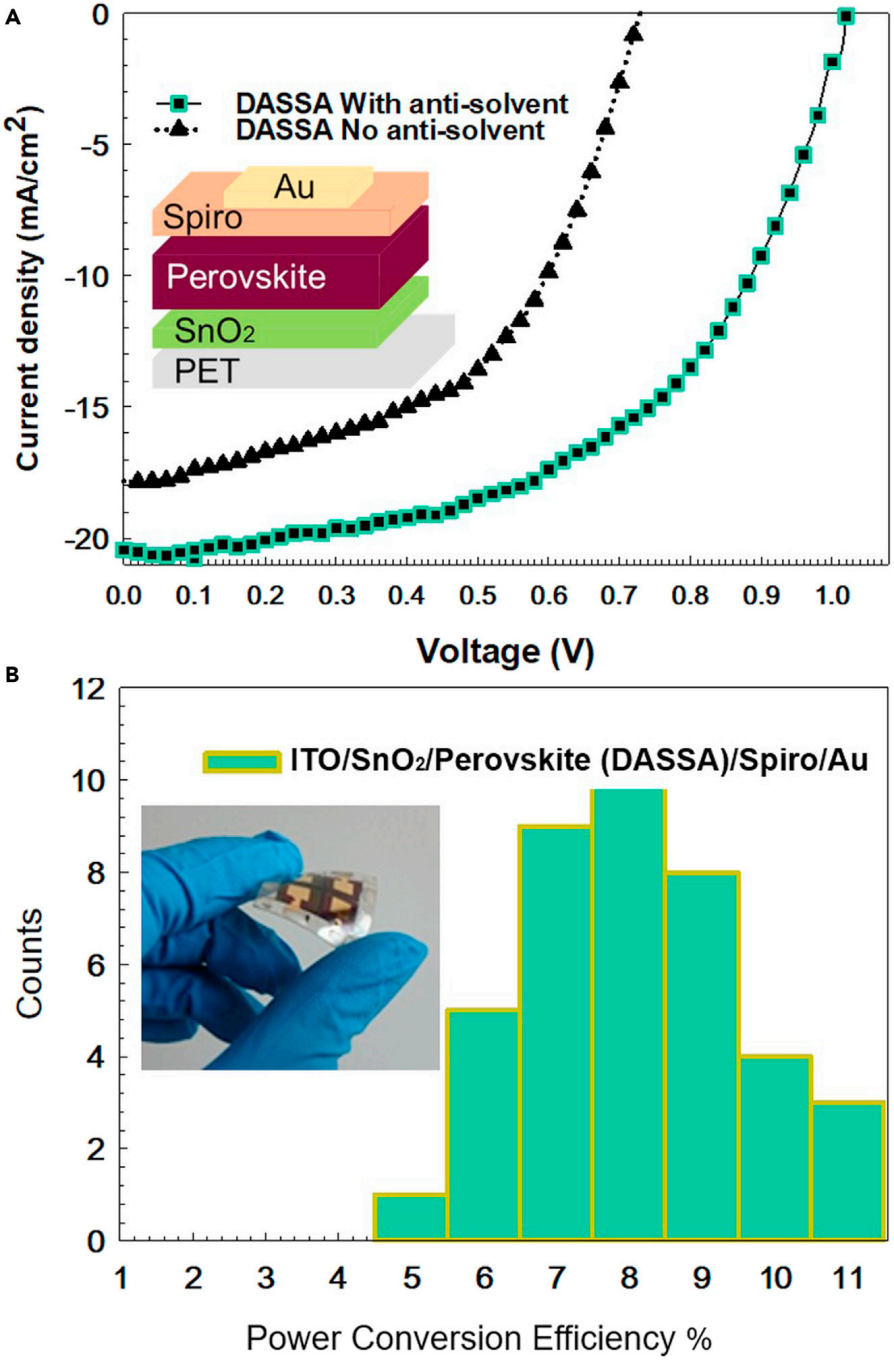
Photovoltaic performance of perovskite solar cells (A) J-V curves of solar cells for which perovskite deposition was carried out via the antisolvent soaked applicator (DASSA) method without and with antisolvent with structure PET/ITO/SnO_2_/perovskite/Spiro/Au, (B) the statistical distribution of the power conversion efficiency (PCE) of 40 DASSA cells prepared with antisolvent.

Figure 6A shows the J−V curves of the devices with PET/ITO/SnO_2_/Perovskite/Spiro/Au architecture. The perovskite film was deposited by DASSA without and with antisolvent at 125°C. The power conversion efficiency PCE of the best performing solar cell with the dry applicator was 6.8%. When antisolvent was added to the paper applicator all PV parameters rose considerably resulting in a remarkable enhancement in average efficiency of 83% in relative terms, with the absolute PCE obtained equal to 11.1%. Table 1 reports the average photovoltaic parameters over 9 flexible cells for DASSA without antisolvent and 40 cells with the antisolvent process.

**Table 1:** Average value, standard deviation, and best cell PV parameters of devices made by deposition via antisolvent-soaked applicator deposition (DASSA), in which the applicator is paper without (dry) and with (i.e. soaked in) antisolvent

Type	VOC(V)	JSC (mA/cm2)	FF	PCE(%)
Deposition with dry piece of paper	0.784 ± 0.097	14.52 ± 2.27	40.8 ± 7.4	4.77 ± 1.50
Best cell deposited with dry piece of paper	0.729	17.83	52.27	6.79
DASSA (with antisolvent)	0.876 ± 0.033	19.32 ± 1.80	48.04 ± 6.05	8.11 ± 1.481
Best cell deposited with DASSA	1.020	20.44	53.3	11.12

Averages were calculated over 9 cells for DASSA without antisolvent and over 40 cells for DASSA with antisolvent.

The external quantum efficiencies (EQE) of the DASSA solar cell were also investigated in the 300-850 nm range with peaks reaching 87% (Figure S3). The calculated integrated photocurrent density was within 5% compared to that measured under one sun. In Figure 6B, we show the reproducibility of the PCE values histogram from 40 devices manufactured with DASSA. Around two-thirds of the devices were able to achieve PCEs of over 8% with the best surpassing 11%. Considering that the application of the paper applicator was carried out manually, and that these are flexible cells on PET (Figure S4), the reproducibility is surprisingly high demonstrating the effectiveness of the method.

**Figure 7:**
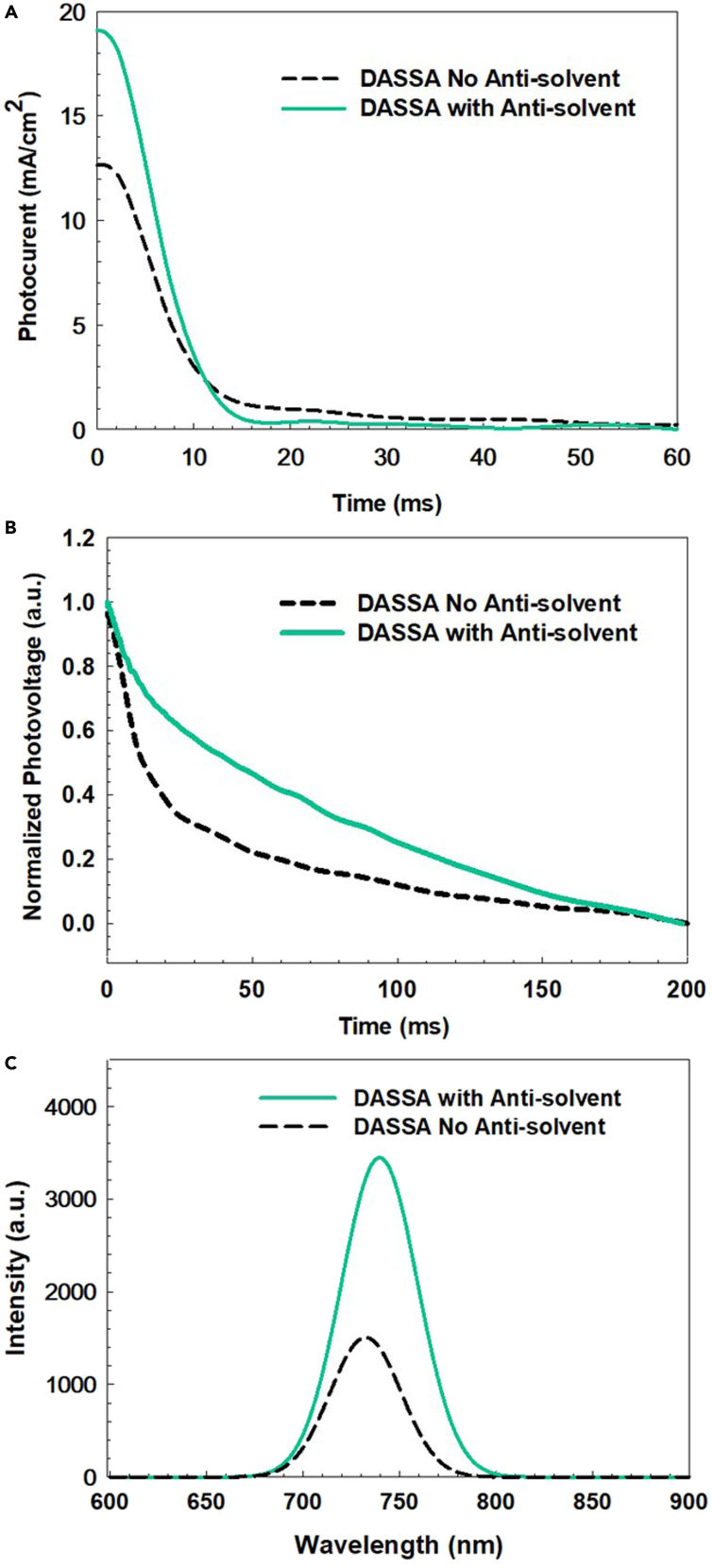
Transient photocurrent (TPC), normalized transient photo-voltage (TPV) and Photoluminescence spectra of the DASSA perovskite films (A) and (B) Transient photocurrent (TPC) and normalized transient photo-voltage (TPV) of PET/ITO/SnO_2_/perovskite/Spiro/Au cells with perovskite layer deposited via an antisolvent soaked applicator method (DASSA) without and with antisolvent. (C) Photoluminescence spectra of the DASSA perovskite films with (continuous green line) and without (dashed black line) Antisolvent soaking of the paper applicator.

We investigated the transient photocurrent (TPC) and the transient photo-voltage (TPV) decay of cells (Figures 7A and 7B) upon and after excitation with a short light pulse. The latter gives information about extracted charges under short circuit conditions. The charge-transport time for the DASSA device deposited with antisolvent (with fall time of 10.3 μs) shows faster charge transport than the DASSA without antisolvent device (with a value of 11.9 μs). TPV measurements relay the recombination rate of carriers, from which the charge carrier lifetime can be estimated. A slow TPV decay can be explained by lower charge recombination rates (Kakavelakis et al., 2018). As shown in Figure 7B, it was found that the charge-recombination lifetime of the DASSA device (with fall time of 135 μs) was much longer than that of the DASSA without antisolvent device (with fall time of 21 μs). The reduced charge recombination is promoted by optimized morphology such as interconnected grains and a low number of pinholes as supported by the SEM/XRD images in the previous section as well as previous literature on transient measurements (Yu et al., 2016; Zarabinia et al., 2021; Tailor et al., 2020).

Figure 7C shows the steady-state PL spectra of the perovskite layers on non-conductive glass. The PL intensity of the DASSA perovskite film is more than double than that without antisolvent. The more intense peak confirms the improved electronic quality of the perovskite film with lower density of non-radiative traps when soaking the paper applicator in antisolvent (Zhang et al., 2015). Future experiments such as temperature dependent PL (Zhang et al., 2020b) and transmission electron microscopy could add further information on the inner structure of the sample. For more insight on the electronic structure of the perovskite films, we performed a self-consistent optoelectronic simulation (involving solution of the Poisson equation and drift-diffusion model with SCAPS) to estimate the density of defect states (taken as fitting parameter) of the perovskite films (see Figure S5 and Table S2). DASSA films have a significantly lower trap density of 3 × 10^16^ cm^−3^ compared to DASSA without antisolvent (8 × 10^16^ cm−^3^). Table S3 reports the PV parameters of the DASSA with antisolvent and without antisolvent devices extracted from both forward and reverse voltage sweeps. The hysteresis index was 0.26 for the device without antisolvent soaking, whereas it was considerably smaller, i.e., 0.18 when the applicator was soaked in antisolvent prior to film deposition, confirming the higher quality of the latter films (Dunbar et al., 2017).

11.1% is a satisfying efficiency, particularly for flexible solar cells on PET and with a totally manual deposition process. Nevertheless, the corresponding PCE achieved with spin coating devices with the antisolvent method was 14.9% (Table S4), although this was carried out in a glove-box rather than in air. SEM images of spin coated films (Figure S6) show more visible crystal-grain morphology, in part resulting from processing in N_2_ environment, (Calabrò et al., 2018) with average sizes of around 500 nm. Spin-coated films also have lower trap densities (2 × 10^15^ cm^−3^) from simulations (Table S2). Setting up an automated system that would carry out the application more precisely compared to the manual deposition we implemented here would improve macroscopic and microscopic film uniformity as well as performance and reproducibility.

**Figure 8:**
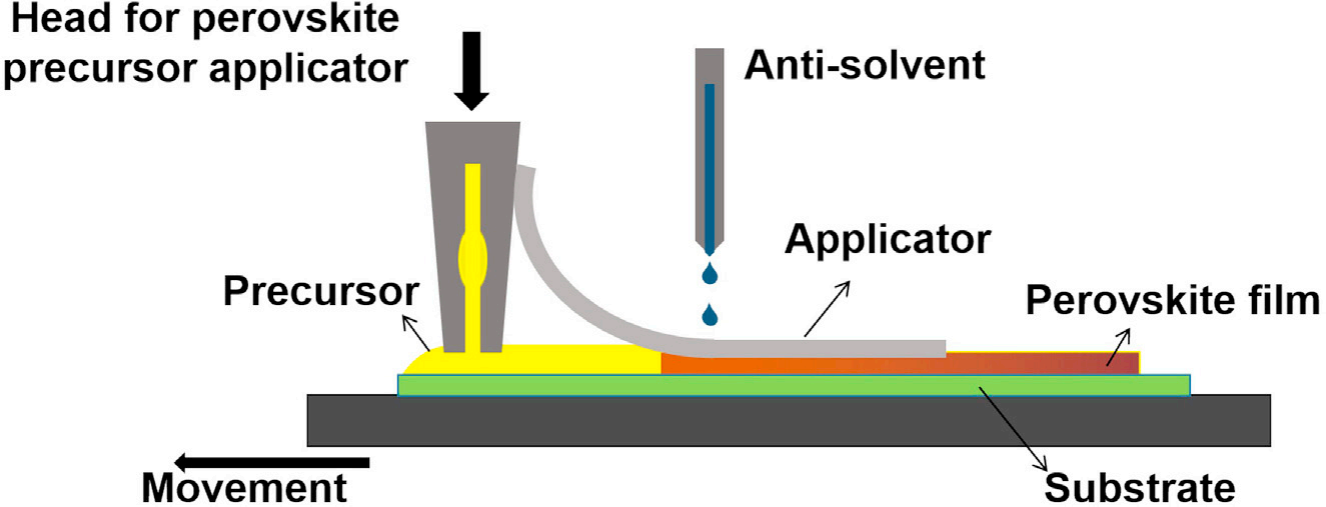
Schematic showing a potential implementation of DASSA (deposition via an antisolvent soaked applicator) for automated industrial purposes

Thus, there is space for further optimization of this new procedure via automation. As illustrated in Figure 8 we envisage attaching a paper sheet applicator (or other similar material) to a blade or slot die coating head soaked in antisolvent. To avoid precursor/solvents accumulating on the applicator over time, a roll of paper that unwinds can be envisaged for the automated method. In this case, the addition of a stream of antisolvent that soaks the applicator can be continuously implemented during deposition. By tuning the parameters of the deposition, as well as the amount of antisolvent on the trailing soft applicator, further improvements can be determined.

### Conclusion

In conclusion, we have successfully introduced a novel deposition technique to deposit high-quality perovskite films in ambient air based on a piece of paper as an applicator, even at low temperatures compatible with plastic substrates. Flexible perovskite solar cells, fabricated at temperatures ≤125°C with this technique delivered a power conversion efficiency of 6.7% with a dry applicator. When the paper applicator was soaked in the green antisolvent, ethyl acetate, the maximum PCE jumped to 11.1%. By soaking the applicator with antisolvent, double cation perovskite films with increased uniformity, crystallinity, and better-interconnection were obtained as evidenced by analysis of the PL, SEM, and XRD measurements. The latter also shows a better compositional structure for the DASSA films. In addition, photovoltage and current transients support J-V simulations which quantify a significantly lower concentration of defects for the films deposited utilizing a paper applicator soaked in antisolvent compared to those deposited with a dry applicator. The new procedure, which enables the formation of pinhole-free uniform polycrystalline perovskite films, opens up fabrication of perovskite layers not only to those labs that do not have any solution processing/deposition technique available but is also conducive to being fully automated for high throughput manufacturing over large areas paving the way for a completely new rapid industrializable deposition method based on soaking the applicator in antisolvents.

### Limitations of the study

Here, a simple and effective deposition method using a paper applicator for perovskite films is demonstrated at low temperatures to manufacture flexible perovskite solar cells. By soaking the piece of paper in antisolvent before application, solar cell performance increases very significantly. We have carried out SEM microscopy, photocurrent transients, photo-voltage decay transients, PL, XRD, and simulations that quantify the density of trap states to compare and gauge the quality of the perovskite films with and without soaking the paper applicator in antisolvent. Other methods that would add further information on film quality, especially in the bulk, would be transmission electron microscopy, temperature-dependent PL, and rocking XRD (in addition to 2Theta) and these could be used to improve the quality of the film further in future studies. In addition, this method has the potential to be fully automated. Suggestions for automatizing the procedure have been presented for future development.

## STAR★Methods

### Key resources table

**Table:** 

REAGENT or RESOURCE	SOURCE	IDENTIFIER
**Chemicals, peptides, and recombinant proteins**		

Lead (II) iodide (PbI_2_)	TCI	CAS: 10101-63-0
L ead (II) Bromide (PbBr_2_)	TCI	CAS: 10031-22-8
Formamidinium iodide (FAI)	Greatcell solar	CAS: 879643-71-7
Cesium iodide (CsI)	Alfa-Aesar	CAS: 7789-17-5
N,N-dimethylformamide (DMF)	Sigma-Aldrich	CAS: 68-12-2
Dimethyl sulfoxide (DMSO)	Sigma-Aldrich	CAS: 67-68-5
Spiro-MeOTAD	Sigma-Aldrich	CAS: 207739-72-8
Tin(IV) oxide (SnO_2_)	Alfa-Aesar	CAS: 18282-10-5

**Software and algorithms**		

SCAPS (Solar Cell Capacitance Simulator)	Marc Burgelman SCAPS 3.3	https://marc-burgelman-scaps.software.informer.com/3.3/

### Resource availability

#### Lead contact

Further information and requests for resources should be directed to and will be fulfilled by the lead contact, Professor Thomas M. Brown (thomas.brown@uniroma2.it).

#### Materials availability

This study did not generate new materials.

### Methods details

#### Experimental procedures

##### Precursors for double cation perovskite

The double-cation perovskite used in this work was FA_0.83_Cs_0.17_PbI_2.2_Br_0.8_ (Li et al., 2020).The precursor solution was prepared by weighting CsI (44 mg/mL from sigma Aldrich), PbI_2_ (277 mg/mL from TCI), PbBr_2_ (147 mg/mL, TCI), FAI (143 mg/mL) in DMF: DMSO (4:1 v:v ratio, Sigma Aldrich). All substances were used as received. The precursor solution was agitated overnight by magnetic stirring.

##### Device fabrication

Bottom electrode/electron transport layer preparation: PET/ITO (Flexvue LRS15 sourced from Eastman) with 125 micron thickness and 15 ohm/square sheet resistance were patterned via laser ablation, cleaned in an ultrasonic bath (5 min in isopropanol and 10 min in deionized water. with each step repeated twice) and UV-irradiated for 15-20 min prior deposition of the SnO_2_ electron transport layer (ETL). For the compact ETL, 200 mL of a SnO_2_ nanoparticles dispersion (15% colloidal dispersion in H2O from Alfa Aesar) was spin coated in air (6000 rpm for 35s), followed by thermal annealing at 100°C for 1 h.

##### Perovskite deposition by spin coating and by antisolvent soaked applicator

For the deposition of perovskite films by spin coating, 100 μL of precursor solution were coated on SnO_2_ at 1500 rpm for 10 s then at 5000 rpm for 20 s. Four seconds before the end of the process, we added 500 mL of ethyl acetate as an antisolvent. Samples were then annealed at 100°C for 10 min.

To produce perovskite films with the DASSA technique, we prepared 20 μL precursor solution of perovskite and 150 μL of ethyl acetate. The applicator was a with 2.5 × 6 cm^2^ piece of paper cut out of a sheet (BM 02, woodfree paper from Guangdong China, 70 g/m^2^ grammage, tensile strength MD ≥ 2.5 KN/m, Bendtsen smoothness ≥40 mL/min). We separate the procedure in three essential steps (i, ii, iii) all of which together last around 20–25 s:

(i) We placed the PET/ITO substrate on a hotplate. Two parallel 50 μm thick strips of Kapton tape were applied to the edges of substrate to keep it attached to the hotplate as well as acting as separators. The gap between the two stripes of tape (∼2 cm) was smaller than the width of the paper applicator so that the latter would rest on the two tape segments and act as separators when the applicator was going to be drawn over the substrate. 5-6s after placing the taped substrate on the hotplate, we soaked the paper sheet in antisolvent. We applied 15 μL of ethyl acetate over 16 cm^2^ of the paper applicator with a pipette. This represents around one-third of the area of the paper applicator (i.e. the part that will come in contact with the precursor film). Then we waited 8s before using the drawing the piece of paper over the wet perovskite precursor (Figure S7).
(ii) During these 8 s, we poured the perovskite precursor on the substrate (between the gaps of the tape stripes, with a 2 × 2.5 cm^2^ area) with a pipette.
(iii) Then we covered the perovskite precursor solution with the paper applicator and pulled the paper applicator from one side to the other (handling the paper applicator on the edge that had not been soaked in antisolvent). The application movement took ∼ 24 s over the substrate (pulling speed of ∼ 0.1 cm/s). The application of the film by moving the paper sheet was carried out as soon as the paper applicator was placed over the substrates, without delay.

We also repeated the same procedure using a Kapton polyimide film from CS Hyde Company (with 25 μ m thickness), tissue (cleanroom wipe) 100% knitted polyester from Hansong company, glossy coated paper (RiteColor G-230) and a piece of dry BM paper (without and with antisolvent) as applicators.

Difference levels of humidity in the paper applicator could lead to uncontrolled chemical reactions during coating. Thus it is important to quantify the moisture content of antisolvent soaked paper sheets for reproducibility. We used a moisture meter (MIC98711 from MIC Meter Industrial Company with moisture range accuracy ∼±1%), at different temperatures (Table S5). The moisture content of paper was 5% at 25°C and decreased to 2.1% at 85°C when soaked with antisolvent in the same conditions used for depositing the perovskite film.

##### Hole transport layer and top electrode preparation

The solution of hole transport layer (HTL) was prepared by dissolving 73.5 mg of Spiro-MeTAD from Sigma Aldrich in1 ml of chlorobenzene and doping with 26.7 μL of 4-tert-butylpyridine, 7.2 μL cobalt (III) complex FK209 (0.25 M in acetonitrile) and 16.6 μL of lithium bis-(trifluoromethyl sulphonyl)imide (LiTFSI) solution (520 mg mL−1 in acetonitrile) (Yu et al., 2016). This solution was deposited by spin coating at 2000 rpm for 20 s in a nitrogen-filled glove box. Finally, we deposited 100 nm of Au (10 nm at 0.3 angstrom/s and 90 nm at 1 angstrom/s) by thermal evaporation through a shadow mask at pressure below 10-6 mbar.

##### Characterization techniques

The morphologies were investigated by a scanning electron microscopy SEM from TESCAN Company and the crystal structural properties were analyzed by X-ray diffraction and X'Pert software. We obtained the photoluminescence (PL) of the prepared cells using a 405 nm laser diode. The current density–voltage (J-V) curves of the flexible perovskite solar cells were measured with a Keithley 2420 source meter under an AM1.5G Class A ABET solar simulator at an intensity of 1000 W/m2 (1 sun) calibrated with an ECO Pyranometer MS-602 at room temperature. The voltage step and scan rate for data point scans were fixed at 20 mV, 100 mV/s, respectively, for each cell measurements in both forward and reverse scan. During the measurement cells were masked with a 0.09 cm2 black tape mask. A UV-Vis 2550 Spectrophotometer from Shimadzu was used to measure the absorbance. We can estimate the thickness of the layer from the absorbance changes with simple relation (α) = 2.303A/t, where (A) is absorbance, (t) is the thickness of thin-film, and (α) is the absorption coefficient. Transient photo-voltage/photocurrent (TPV and TPC), and the incident photon-to-current conversion efficiency (IPCE) were evaluated by using a modular testing Arkeo platform (Cicci Research s.r.l.).

As also shown in our previous studies (Lucarelli and Brown, 2019; Di Giacomo et al., 2016b; Dagar et al., 2018; Lucarelli et al., 2017), flexibility of the perovskite solar cell are limited by the flexibility of the ITO (which has a safe bending radius of 7 mm) rather than by the perovskite or transport layers. New transparent electrodes would need to be implemented for going down to very low curvatures (Lucarelli and Brown, 2019).

##### Simulations

SCAPS simulation was a one-dimensional solar cell simulation program developed at the Department of Electronics and Information Systems (ELIS) of the University of Gent, Belgium. It is freely available to the PV research community (universities and research institutes). SCAPS simulation consists of the solution of drift-diffusion equations and effective parameters as bandgap energy, defect density, electron affinity acceptor/donor concentration, and mobility in the J–V curve.

### Quantification and statistical analysis

We report the average photovoltaic parameters and standard deviations to compare solar cell performance under the same standard test conditions. We also provide a statistical analysis of power conversion efficiency on 40 different samples fabricated with the same procedure.

## Data Availability

• Data: All data reported in this paper will be shared by the lead contact upon request. • Code: This paper does not report original code. • Any additional information required to reanalyze the data reported in this paper is available from the lead contact upon request.

## Supplemental information

Document S1. Figures S1–S6 and Table S1 –S4

Video S1. The visual model of DASSA method, related to Figure 1
