# Supplementary material for: Simple and effective deposition method for solar cell perovskite films using a sheet of paper
Source: iScience. 2021 Dec 31;25(2):103712. doi: 10.1016/j.isci.2021.103712 (PMC8783128; doi:10.1016/j.isci.2021.103712)
Supplement: Document S1. Figures S1–S6 and Table S1 — –S4 [file mmc1.pdf]

iScience, Volume *25*

Supplemental information

Simple and effective deposition method for solar cell perovskite films using a sheet of paper

Nazila Zarabinia, Giulia Lucarelli, Reza Rasuli, Francesca De Rossi, Babak Taheri, Hamed Javanbakht, Francesca Brunetti, and Thomas M. Brown

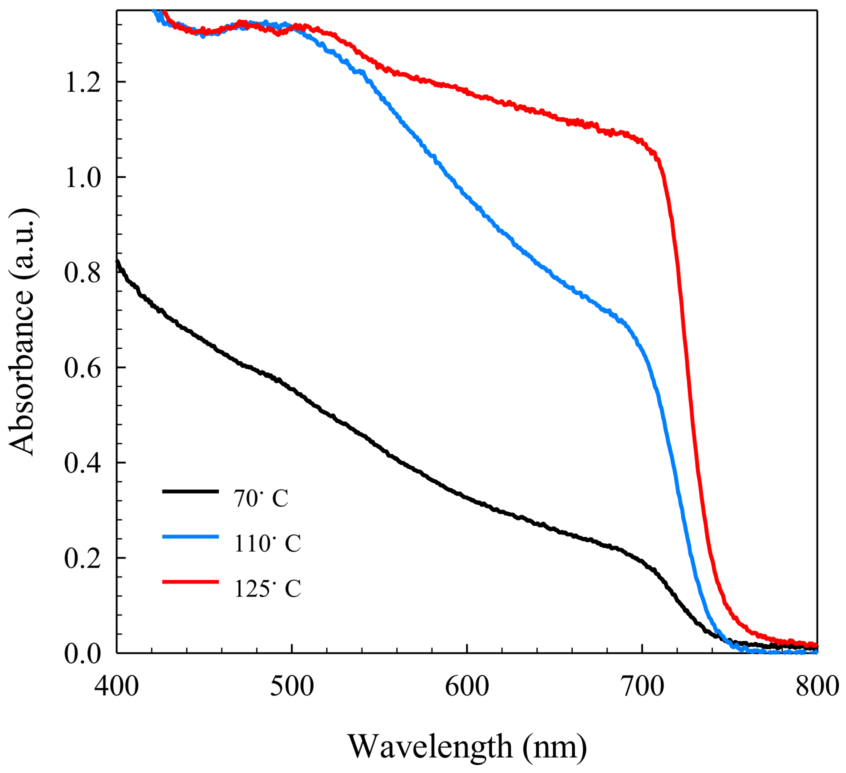

Figure S1. Absorbance spectra of double-cation mixed-halide perovskite film fabricated at various temperatures, related to Figure 3.
UV-Vis absorbance spectra of ITO/SnO_2/perovskite film by DASSA at various temperatures 70°, 110°, and 125°C

The absorbance changes are due to thickness changes following the Beer Lambert low

$I = I_0 e^{(-\alpha t)}$

Where, (t) is thickness of thin film and (α) is the absorption coefficient.

$Log(I/I_0) = -\alpha t \log(e)$, $\log(I/I_0)=A$

So, experimentally, the absorption coefficient (α) can be calculated from this simple relation $\alpha t=A\ (2.303)$.

The absorption coefficient (α) is derived from the quantum-mechanical probably of transition from valance band VB to conduction band CB of material. The absorption coefficient rises with the photon energy following

$\alpha h\nu \approx (h\nu - E_g)^{1/2}$

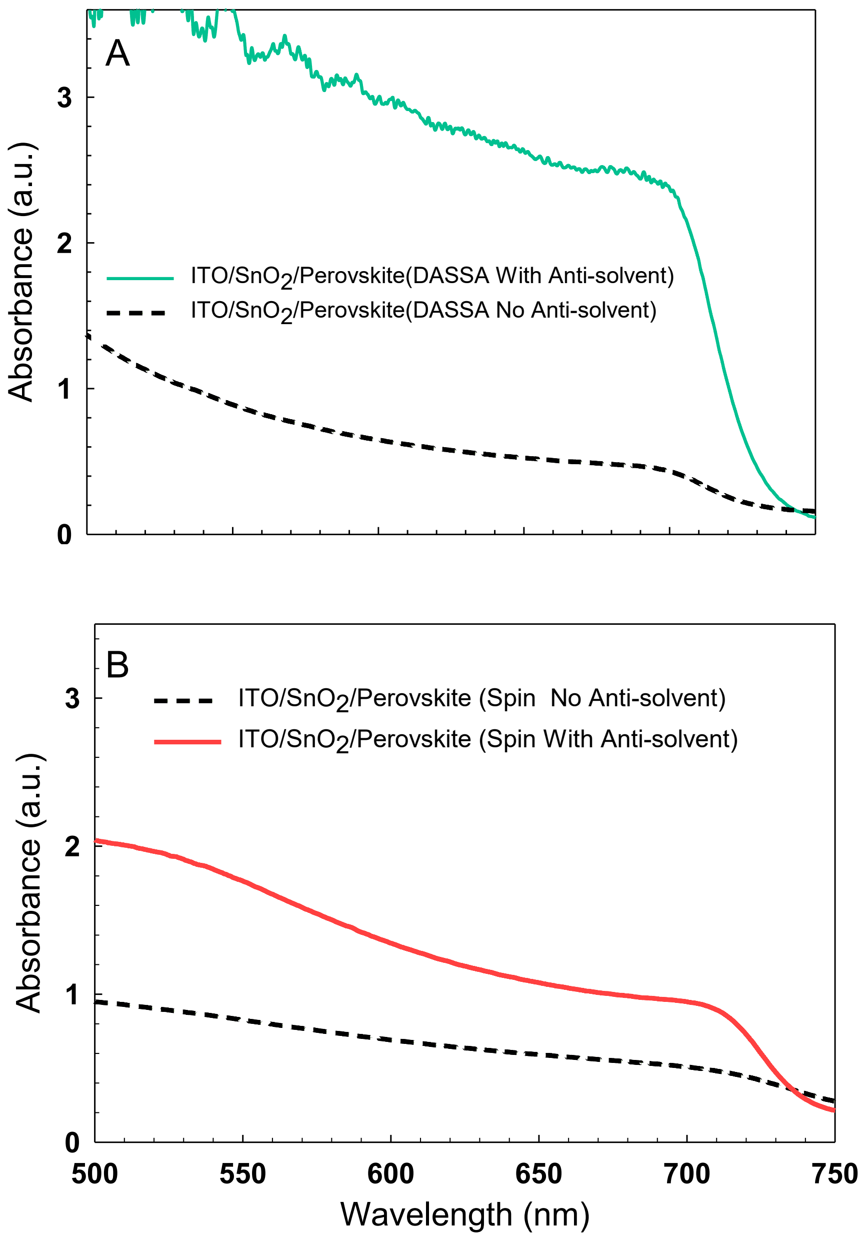

Figure S2. Absorbance spectra of double-cation mixed-halide perovskite film fabricated, related to figure 4.
(A) deposition via an anti-solvent soaked applicator (DASSA) without and with anti-solvent (at 125° C).
(B) spin coating (Spin) without and with anti-solvent. Two samples for every method were fabricated and tested.

The average thickness of the spin coating with anti-solvent film was about 350-450 nm, whereas that by the paper applicator was estimated to be around 580-720 nm using the Beer-Lambert law. The thickness of the perovskite layer estimated from cross-sectional SEM images was 710 nm

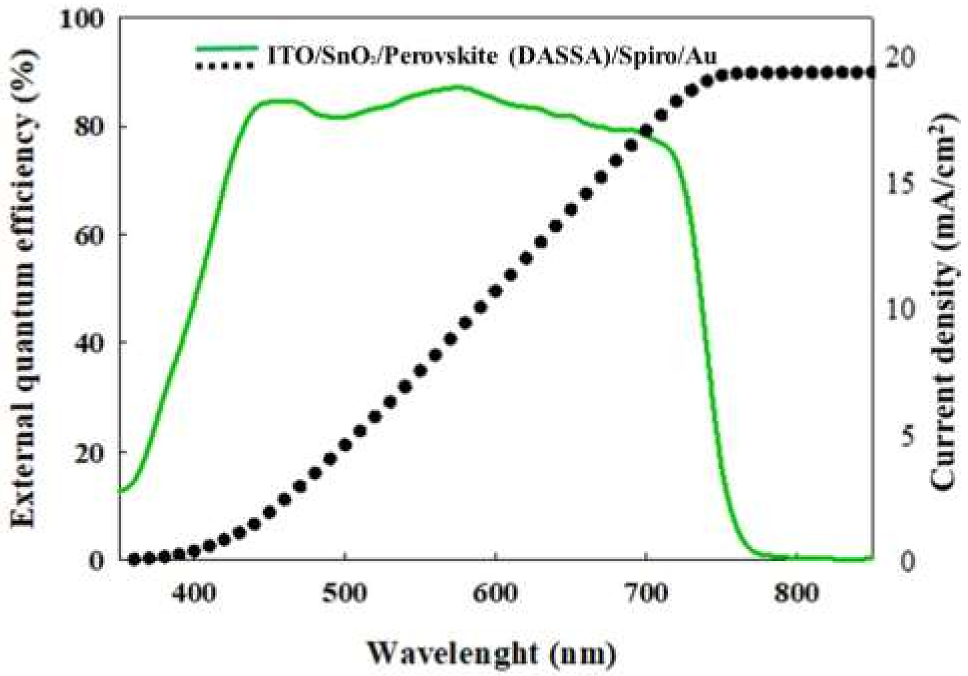

Figure S3. The external quantum efficiency spectrum of device, related to Figure 6.

The device made by deposition via an anti-solvent soaked applicator (DASSA)

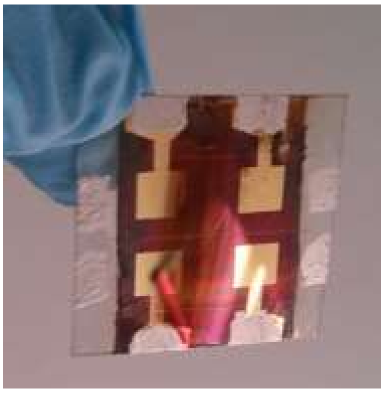

Figure S4. Image of perovskite device, related to Figure 6.

Flexible substrate (2.5×2.5 cm^2) were patterned to create four active area of PET/ITO/perovskite/SPIRO/Au structure for the cell performance measuring (with mask of 0.16 cm^2).

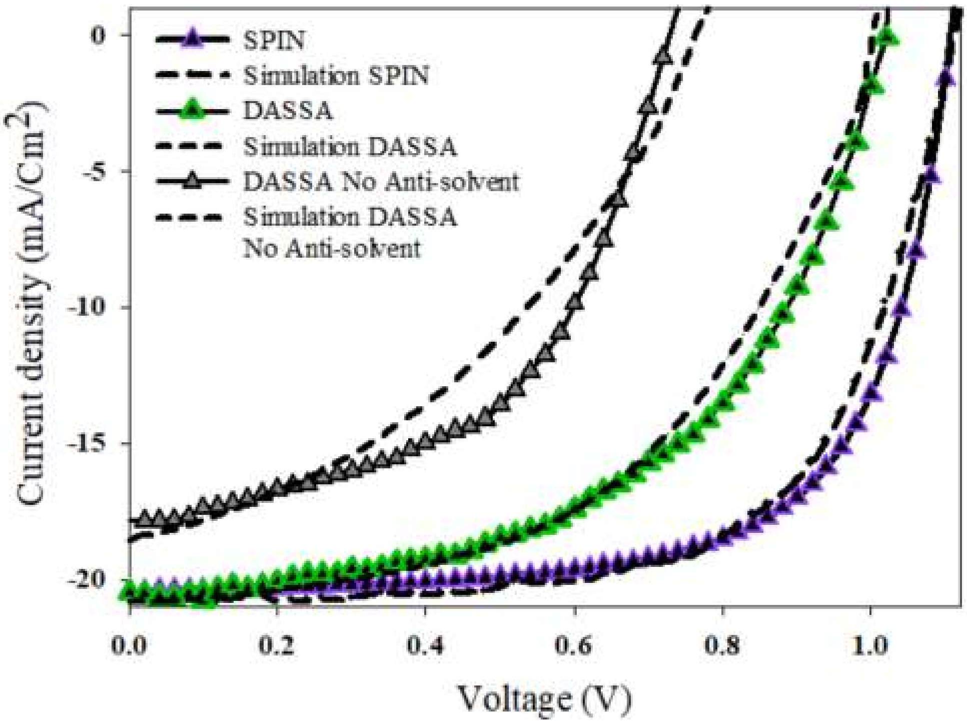

Figure S5. The simulated J-V characteristics for spin coating and DASSA method and DASSA without anti-solvent of the corresponding experimental data, related to Figure 6.

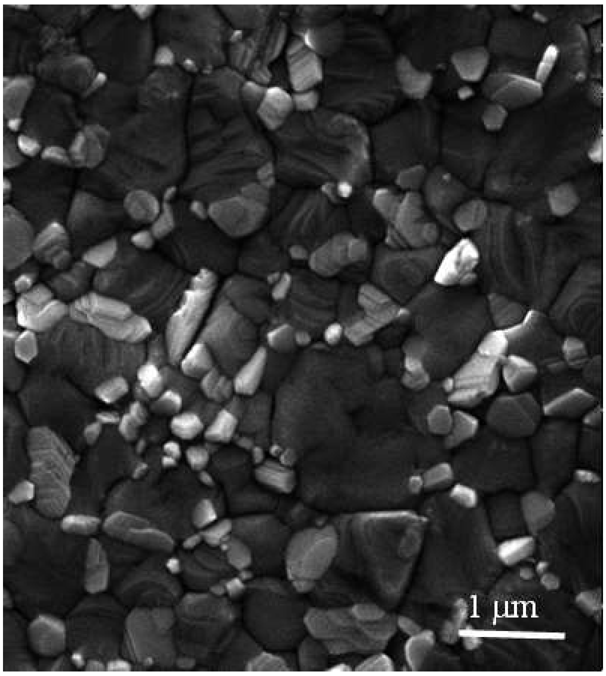

Figure S6. SEM images of spin-coated perovskite films with an anti-solvent, related to Figure 5.

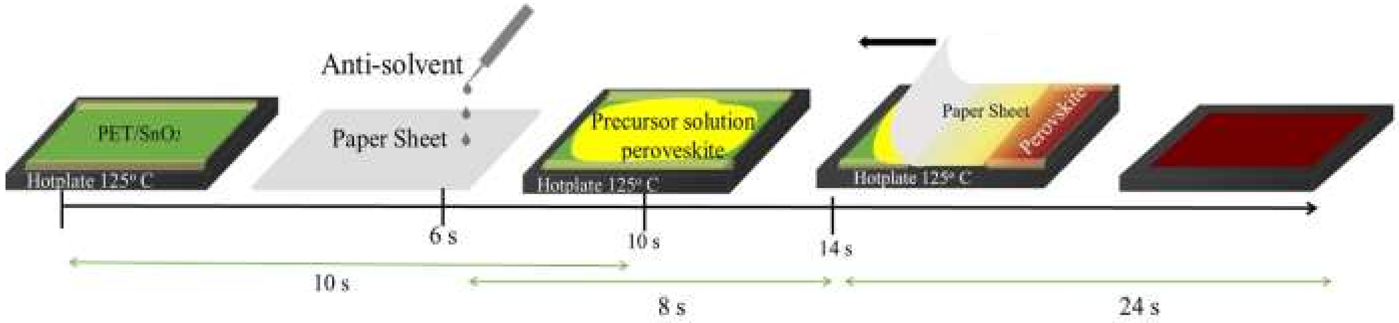

Figure S7. Figure of time processes of experimental part of deposition via anti-solvent-soaked applicator (DASSA), related to Figure 1.

Table S1. Characteristic of XRD peaks for DASSA with and without anti-solvent, related to Figure 5.

It is calculated by Xpert software. The height of 100 peaks for with and without anti-solvent have been normalized.

	Positon(2θ)	Height	FWHM
DASSA	14.14	404	0.28
	20.11	48.3	0.43
	28.61	252.9	0.36
	40.99	67	0.12
DASSA No Anti-solvent	13.99	404	0.36
	19.92	43.5	0.43
	28.38	158.5	0.64
	40.73	58	0.36

Table S2. Parameters of simulated J-V characteristics for spin coating, DASSA method and DASSA without anti-solvent, related to Figure 6.

Parameters	Spin	DASSA	DASSA No Anti-solvent
Thickness (μm)	0.39	0.710	0.730
Band gap energy E_g (eV)	1.6	1.6	1.6
Electron affinity (eV)	3.8	3.8	3.8
Defect density N_t (cm^{-3})	2×10^{15}	3×10^{16}	8×10^{16}
Effective conduction band density N_c (cm^{-3})	1×10^{-19}	1×10^{-19}	1×10^{-19}
Effective valance band density N_v (cm^{-3})	1×10^{-19}	1×10^{-19}	1×10^{-19}

Table S3. Data of J-V characteristics resulting from sweeping the voltage from forward to reverse and from reverse to forward bias together with the hysteresis index, related to Figure 6.

Type of device	Voc V	Jsc mA/cm^2	V_MPP V	J_MPP mA/cm^2	P_MPP mW/cm^2	FF %	Hysteresis index
FW DASSA with Anti-solvent	0.8920	20.2974	0.6000	13.6695	8.2017	45.30	0.18
RV DASSA With Anti-solvent	0.8991	20.8439	0.6600	16.1864	10.6830	57.00	
FW DASSA No Anti-solvent	0.7260	15.8647	0.5200	9.5902	4.9869	43.30	0.26
RV DASSA No Anti-solvent	0.7290	17.8326	0.5000	13.5895	6.7947	52.27	

Table S4. PV parameters of devices made by the spin-coated, deposition with dry piece of paper and deposition via an anti-solvent soaked applicator DASSA, related to Figure 6.

Type	V_{OC}(V)	J_{SC} (mA/cm^2)	FF	PCE (%)
Spin-coated	1.068$\pm$ 0.031	19.20$\pm$ 1.26	63.25 $\pm$ 2.04	12.96 $\pm$1.02
Best cell Spin-coated	1.114	20.92	64.08	14.94
Deposition with dry piece of paper	0.784 $\pm$ 0.097	14.52 $\pm$2.27	40.8$\pm$7.4	4.77 $\pm$1.50
Best cell deposited with dry piece of paper	0.729	17.83	52.27	6.79
DASSA (with anti-solvent)	0.876±0.033	19.32±1.80	48.04±6.05	8.11 ±1.481
Best cell deposited with DASSA	1.020	20.44	53.3	11.12

Table S5. The effect humidity of paper with and without Anti-solvent at different temperature, related to Figure 1.

Temperature	25°C	50°C	85°C
paper (without Anti-solvent) moisture	~ 5%	~ 3.4%	~ 2.5%
anti-solvent soaked paper moisture	~ 3.4%	~ 2.3%	~ 2.1%
